# Uncovering the Male Presence in Parthenogenetic *Marchalina hellenica* (Hemiptera: Marchalinidae): Insights into Its mtDNA Divergence and Reproduction Strategy

**DOI:** 10.3390/insects14030256

**Published:** 2023-03-04

**Authors:** Nikoleta Eleftheriadou, Umar K. Lubanga, Greg K. Lefoe, M. Lukas Seehausen, Marc Kenis, Nickolas G. Kavallieratos, Dimitrios N. Avtzis

**Affiliations:** 1 Laboratory of Agricultural Zoology and Entomology, Faculty of Crop Science, Agricultural University of Athens, 75 Iera Odos str., 11855 Athens, Greece; 2 Agriculture Victoria, Department of Energy, Environment and Climate Action, AgriBio Centre, Bundoora, VIC 3083, Australia; 3 Centre for Agriculture and Bioscience International, Rue des Grillons 1, 2800 Delémont, Switzerland; 4 Forest Research Institute—Hellenic Agricultural Organization Demeter (HAO Demeter), Vassilika, 57006 Thessaloniki, Greece

**Keywords:** Marchalinidae, invasive species, parthenogenesis

## Abstract

**Simple Summary:**

*Marchalina hellenica* (Hemiptera: Marchalinidae) is a significant contributor to annual honey production in Greece and Turkey, where it is endemic. It was initially described as parthenogenetic, producing only females. The exact reproduction strategy of this species remains unknown. For this reason, we studied the emergence pattern of male individuals in Greece for two consecutive years (2021 and 2022). Furthermore, we examined the genetic variation among 15 geographically distant populations of *M. hellenica* in Greece using a mitochondrial DNA marker and compared the results with data from Turkey. This study documents the existence of an additional *M. hellenica* population in its native range that repeatedly produces males, suggesting a previously unknown role for males in the species’ reproduction. The Greek and Turkish populations exhibited a strong genetic affinity, while the genetic pattern in Greece seems to have been obscured by human-aided dispersal.

**Abstract:**

*Marchalina hellenica* (Hemiptera: Marchalinidae), an endemic species in Greece and Turkey, is a major contributor to the annual honey production in its native range. However, in the areas that it invades, lacking natural enemies, it has detrimental effects on pine trees and potentially contributes to tree mortality. Although it was originally reported as thelytokous, males were later reported in Turkey and on several of the islands of Greece. To further disambiguate the exact parthenogenetic reproduction strategy of *M. hellenica*, we studied the emergence pattern of male individuals in Greece for two consecutive years (2021 and 2022). Furthermore, we examined the genetic variation among 15 geographically distant populations of *M. hellenica* in Greece using a mitochondrial DNA marker and compared the results with data from Turkey. The findings of this study document the existence of an additional *M. hellenica* population in its native range that repeatedly produces males, apart from the areas of Greece and Turkey in which they were initially reported, suggesting that males play a major, so far unknown role in the reproduction of this species. The populations in Greece and Turkey exhibited a strong genetic affinity, while human-aided dispersal seems to have obscured the genetic pattern acquired.

## 1. Introduction

*Marchalina hellenica* (Gennadius) (Hemiptera: Marchalinidae), a scale-insect species native to Greece and the coastline of Turkey [1,2,3], is the most significant honeydew-producing insect in Greece [1,4]. It feeds on the sap of pine trees (*Pinus* spp.), excreting a glutinous substance of slightly modified tree sap, called honeydew [1,2,5,6]. In its native range, *M. hellenica* is deemed a key insect for apiculture, since the honeydew produced by the scale is collected by bees, *Apis mellifera* Linnaeus (Hymenoptera: Apidae), and converted to pine honey, representing 60% of the honey production in Greece annually [5,6] and 50% in Turkey [6]. For this reason, there has been a significant concern among beekeepers in recent years, following the observation of a notable reduction in the amount of honeydew [2]. In Greece, *M. hellenica* primarily infests *Pinus brutia* and *Pinus halepensis*, but it has also been found on *Pinus pinea, Pinus nigra, Pinus maritima, Pinus sylvestris* [7,8], and *Abies cephalonica* [6]. Beyond its native range, *M. hellenica* has also been reported on *Pinus leucodermis* and *P. maritima* on the island of Ischia, in Italy [9], on *P. halepensis* and *P. pinea*, in Croatia [10], and on *Pinus radiata*, in Australia [11]. Although, in the past, *M. hellenica* was thought to infest *Picea* sp. in Russia, Armenia, and Georgia [12], it was later determined that the scale-insect species encountered in these countries was *Marchalina caucasica* Hadzibeyli (Hemiptera: Marchalinidae) [13]. In its native range, *M. hellenica* is not considered a serious pest and control measures are taken only sporadically, mainly for aesthetic reasons in urban areas [14]. Although *M. hellenica* is associated with detrimental effects on trees at high densities, such as branch and foliage desiccation, growth decline, and crown transparency [15,16], it only rarely causes tree mortality, and usually only in conjunction with other biotic and abiotic secondary stress factors [15,16]. In regions invaded by *M. hellenica*, similar or greater impacts on host trees have been observed [14]. The mild adverse effects of *M. hellenica* on pine trees in its native region have been attributed to the impact of its natural enemies [11]. In particular, *Neoleucopis kartliana* (Tanasijtshuk) (Diptera: Chamaemyiidae) is considered to be the most important natural enemy of *M. hellenica*, suppressing its populations in Greece [11], and it has been successfully used for the biological control of *M. hellenica* on the island of Ischia, Italy [17]. The recent invasion of *M. hellenica* in Australia triggered further studies on the biology of *N. kartliana* [18] and its prospects as a biological control agent against *M. hellenica* in that country [11].

*Marchalina hellenica* is univoltine and undergoes three female and four male nymphal instars [1,13]. Adult females, which bear 11-segmented antennae and lack mouthparts, usually appear on the branches of pine trees during April, where they oviposit a mean of 262 eggs in woolly ovisacs [1,2,5]. The 1st-instar nymphs, which bear 6-segmented antennae and have proportionately enlarged mouthparts, are encountered on trees between late April and early May, where they settle in groups inside bark crevices [1,2]. In early September, the 2nd-instar nymphs, which also bear 6-segment antennae and large mouthparts, appear on the trees [1,2]. In October, the nymphs molt into their 3^rd^ instar, and overwinter until they molt again in April and give rise to adult females [1,2]. Third-instar female nymphs bear 9-segmented antennae and are significantly larger than 1st- and 2nd-instar nymphs. Although females are apterous [13], they can disperse to adjacent trees by walking and their ovisacs can be easily carried away by the wind [5].

There are three main insect genetic reproduction systems, diplodiploidy (with diploid males), haplodiploidy (with effective haploid males), and thelytoky (with no males) [19]. Based on the occurrence of parthenogenesis, parthenogenetic systems are categorized as either facultative, obligate, or cyclic [20]. Depending on the sexes produced by parthenogenesis, it is classified as arrhenotoky (producing only males), thelytoky (producing only females) and amphitoky or deuterotoky (producing both sexes) [20]. Mixed systems involving cyclic or facultative parthenogenesis can occur by switching between thelytoky and either diplodiploidy or haplodiploidy [19]. Most scale-insect families belong to a monophyletic clade that exhibits paternal genome elimination [21,22], and they exhibit a wide range of genetic systems [23], with parthenogenesis being either thelytokous, deuterotokous, or arrhenotokous [24]. Nur [25] described six parthenogenetic systems observed in scale insects based on (a) whether male individuals are absent or appear occasionally (obligate parthenogenesis and facultative parthenogenesis, respectively), (b) which sexes are produced by fertilized and non-fertilized eggs, and (c) how diploidy is restored in non-fertilized eggs [26]. There are only a few known obligatory thelytokous scale-insect species, e.g., *Protopulvinaria pyriformis* Cockerell (Hemiptera: Coccidae), and *Pulvinaria peregrina* (Borchsenius) (Hemiptera: Coccidae), which do not produce males in any geographic region [27]. Although many species were initially considered thelytokous [25], they were later observed to produce males amphimitically or parthenogenetically [24]. *Marchalina hellenica* was originally reported as obligatory thelytokous, since males were considered absent [25,28] and its females had no spermatheca [29]. Nikolopoulos [30] and Minachilis [31] first described males that were thought to belong to *M. hellenica*. However, it was later revealed that they belonged to a *Palaeococcus* (Hemiptera: Margarodidae) species [2,32]. In the early 2000s, Hodgson and Gounari [13] described apterous *M. hellenica* males, which have since been reported only on Greek Aegean islands (e.g., Rhodes, Crete, Samos, Ikaria) [2] and in Muğla province in Turkey [33]. Little is known about the exact role of males in the reproduction of *M. hellenica* and the circumstances under which they emerge.

Although studies have been conducted on the biology of *M. hellenica* in recent decades [1,4,5,13,33], the exact reproduction system of *M. hellenica* and its relation to genetic divergence remain largely unknown. Its population performance and reproduction system should be considered to estimate the evolution of a potential or ongoing invasion, since parthenogenetic species are commonly invasive [34]. Most of the genetic diversity seen in asexual arthropod populations could arise from multiple origins of clones from sexual ancestors rather than mutations within the asexual population [35,36]. Provided that *M. hellenica* is considered mainly parthenogenetic, an interesting question is whether different or geographically distant populations of *M. hellenica* are genetically divergent. This question has concerned the research community in the past. For instance, Bouga et al. [37] revealed a genetic population homogeneity of *M. hellenica* between Greece and Turkey, exhibiting only one haplotype in their mtDNA analysis. Thus, the objective of this research is to investigate the emergence pattern of male *M. hellenica* individuals and examine the genetic variation among geographically distant populations in Greece by using mtDNA markers, comparing them to already existing sequences deposited in GenBank. Through this approach, we intend to elucidate the intricate reproduction strategy of *M. hellenica* and gain a better understanding of its ecology in invaded areas.

## 2. Materials and Methods

### 2.1. Genetic Structure of Marchalina hellenica in Greece

To investigate the genetic variation among geographically distant populations of *M. hellenica* in Greece, samples of female individuals were collected from 13 populations of continental Greece (Katerini, Makriyalos, Alexandroupoli, Stratoni, Thessaloniki, Ioannina, Parga, Athens, Patra, Megalopoli, Korinthos, Larissa, and Kavala) and from two Greek islands (Samothraki and Lefkada). DNA was extracted from 113 *M. hellenica* individuals originating from the aforementioned populations using PureLink™ Genomic DNA Mini Kit (ThermoFisher Scientific, Life Sciences Solutions, Waltham, MA, USA) following the protocol suggested by the manufacturer. The DNA barcoding was then performed in volumes of 25 μL with HCO/LCO primers that amplify a fragment of mtDNA COI gene (654 bp) [38] and MyTaq™ Red Mix (BioLine GmbH, Luckenwalde, Germany). The PCR amplification consisted of an initial denaturation step of 5 min at 94 °C, followed by 5 cycles of 60 s at 94 °C (denaturation), 75 s at 47 °C (annealing), and 90 s at 72 °C (extension). This loop was then followed by 40 cycles of 60 s at 94 °C, 75 s at 52 °C (annealing), and 90 s at 72 °C (extension). The final extension period was performed at 72 °C for 7 min. Purification of PCR products was performed with PureLink™ PCR Purification Kit (ThermoFisher Scientific, Life Sciences Solutions, Waltham, MA, USA) following the protocol of the manufacturer. Sequencing was performed at CEMIA SA (Larissa, Greece) using a sequencer ABI 3730XL. Obtained sequences were examined manually using Chromas Lite software version 2.01 and then blasted in NCBI GenBank. To map the distribution of the obtained haplotypes, visualization was conducted using the QGIS 3.28.2 software based on the World Geodetic System 1984 (WGS 84) [39].

### 2.2. Biological Traits of Marchalina hellenica Males

For the study on the occurrence of male individuals of *M. hellenica*, branch samples of *P. brutia* infested by the scale were collected every 15 days for two consecutive years from the suburban forest of Thessaloniki (Kedrinos Lofos), in northern Greece. Branches with perimeters ranging from 2 cm to 13 cm and lengths ranging from 5.5 cm to 62.5 cm were selected using a measuring tape (DSOMHZ, length 150 cm, accuracy 1 mm), collected using extended pruners (Stanley Garden BDS6311), and individually placed in labeled plastic bags. Samples were transferred to the Laboratory of Forest Entomology (Forest Research Institute, HAO Demeter) at Thessaloniki (Greece), where they were studied under a stereomicroscope (Zeiss Stemi 508, Germany, 6.3–50× magnification range) to detect and isolate male *M. hellenica* adults. *Marchalina hellenica* individuals (min = 100) were also isolated on every collection day to estimate their developmental stage according to the descriptions of Hodgson and Gounari [13]. Since sex determination is not yet feasible in 1st and 2nd *M. hellenica* instar nymphs [13], the developmental stage of the early instars of the scale insect was estimated regardless of sex. The 3^rd^-instar female nymphs and adults of *M. hellenica* females, as well as the 4^th^ instar and adults of *M. hellenica* males were recorded. The developmental-stage determination of females is considered crucial to estimate the emergence of male individuals in relation to females. Finally, the samples were transferred in ventilated cages (60 × 60 × 60 cm) in field conditions to record and collect any male adults that might have emerged. The cages were examined daily. The date and number of any emerging male *M. hellenica* individuals were recorded. Male adults were initially detected visually, since they have elongated bodies and dark legs and antennae [13], and then collected and kept in 98% ethanol. Subsequently, the identification of males was conducted based on the descriptions of Hodgson and Gounari [13] using a stereomicroscope.

#### Statistical Analysis

The association between the developmental stage of *M. hellenica* and the emergence of male adults was analyzed with a quasi-Poisson generalized linear model using the glm function in R [40]. A quasi-Poisson distribution was assumed because the Poisson distribution returned overdispersed residuals. The developmental stage of *M. hellenica* was considered as the independent variable and the count of emerging adults as the dependent variable. To determine which *M. hellenica* female instars are significantly associated with the male counts, a post hoc test with Tukey adjustments was performed.

## 3. Results

### 3.1. Genetic Structure of Marchalina hellenica in Greece

**Figure 1:**
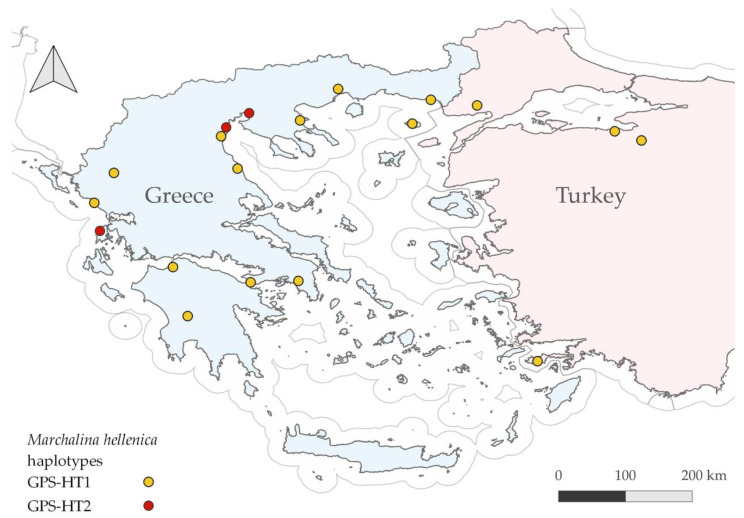
Haplotypes revealed by the mtDNA analysis in Greece (this study) and Turkey [37]. Haplotype 1 (GPS-HT1, yellow points) dominates Greece and four sites in Turkey, while haplotype 2 (GPS-HT2, red points) is exhibited only in three sites in Greece (Thessaloniki, Makriyalos, and Lefkada).

Out of the 113 *M. hellenica* sequences obtained, only two haplotypes were retrieved. These haplotypes differed only by a single nucleotide polymorphism (SNP), between cytosine (C) and thymine (T). The haplotype bearing cytosine (GPS-HT1, GenBank accession OQ506006) was identical to the GenBank accession HQ225738 that was identified by Bouga et al. [37] in four Turkish populations. Most of the individuals from the 15 Greek populations (94/113) exhibited the haplotype GPS-HT1, with only 19 out of the 113 individuals having the mutation that ranked them to the second haplotype (GPS-HT2, GenBank accession OQ506007). All the analyzed individuals from Thessaloniki, Makriyalos, and the island of Lefkada belonged to the rarer haplotype, GPS-HT2 (Table 1 and Figure 1), whereas all the remaining individuals from the other locations in Greece belonged to GPS-HT1 (Figure 1). The two haplotypes obtained in this study were not found simultaneously in any of the 15 sites studied. At each site, all the specimens exhibited a single haplotype (GPS-HT1 or GPS-HT2).

### 3.2. Biological Traits of Marchalina hellenica Males

**Figure 2:**
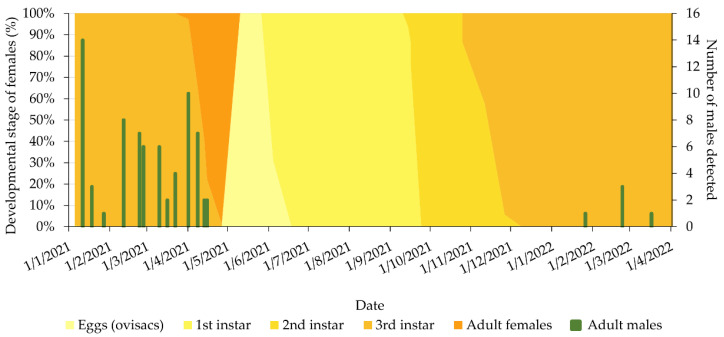
Percentage of the developmental stages of *M. hellenica* females (colored areas) and emergence of *M. hellenica* males (columns) in Kedrinos Lofos (Thessaloniki) between January 2021 and April 2022.

Male *M. hellenica* individuals matching the descriptions of Hodgson and Gounari [13] were encountered in the samples from Thessaloniki both in 2021 and 2022. In 2021, a total of 70 *M. hellenica* males were found roaming inside the cages, while 2 additional adult males were found directly on the regularly collected *M. hellenica*-infested branches during the examination. Adult males were detected from early January to mid-April, when 3^rd^-instar female nymphs and adult females were present (Figure 2). In 2022, male *M. hellenica* adults were again detected inside the cages in which the *M. hellenica*-infested branches were kept, in identical conditions to those in 2021, although in much lower numbers and with a shorter emergence duration. A total of 5 *M. hellenica* males were detected from late January to late March 2022 (Figure 2). It is worth noting that all the males encountered during this study were highly mobile inside the cages compared to the roaming females.

The emergence of males was significantly related to the female developmental stages (χ^2^ = 16.251; df = 4,63; *p* =0.0027). In that, males only emerged concurrent with the 3^rd^-instar nymphs (mean = 1.7 males per week) and adult females (mean = 3.7 males per week) and not during any of the other developmental stages (Figure 2).

## 4. Discussion

### 4.1. Genetic Structure of Marchalina hellenica in Greece

It is generally believed that parthenogenetic lineages are likely to suffer early extinction [41,42] because of the genetic bottlenecks that occur during the onset of parthenogenesis [43]. However, the ability to reproduce asexually facilitates the settlement of a species in a new area, because a single female individual can establish a new population [44,45,46,47]. Parthenogenesis is one of the most effective processes to overcome low population levels and low genetic diversity through uniparental propagation. This assists the expansion of a given species and the exploitation of resources [48]. Indeed, founder populations are typically restricted in size; in addition, parthenogenetic species do not need to find mates and, therefore, do not suffer from inbreeding in the manner of sexually reproducing species [49]. Additionally, parthenogenesis is likely to weaken the Allee effect and favor invasiveness [50]. The low migratory ability and the reproduction strategy of *M. hellenica* are the main characteristics that should be considered in population genetic studies. Both male and female *M. hellenica* adults are apterous [13]; therefore, their natural dispersal ability is considered low, and the main reproduction strategy of the species is parthenogenesis [5]. Due to these features, *M. hellenica* is not expected to exhibit high genetic variation [37]. Intraspecific variation in parthenogenetic organisms is attributed to different sources of parthenogenesis [51], through repeated hybridization and/or polyploidy [52,53], while many parthenogenetic species exhibit high genetic diversity, which can potentially compensate for the absence of DNA recombination [54]. Considering that the mitochondrial DNA (mtDNA) of eukaryote cells has a fast mutation rate, estimated to be 10–20× higher than that of nuclear DNA [55,56,57], leading to significant variation in mtDNA sequences, mtDNA markers have been extensively used to address evolutionary and population questions [37]. In asexual species, DNA recombination is usually insignificant, and such species are expected to have a low mutation rate due to the cost of replication fidelity and deleterious mutations [58]. Furthermore, it has been reported that asexual organisms accumulate deleterious mutations quicker than sexual organisms [59]. By contrast, the asexual and polyploid lineages of some tetrapods exhibit heteroplasmy and mtCOI changes more frequently than the sexual lineages [60,61]. Heteroplasmy (the occurrence of two or more mtDNA variants within a cell) is considered to rise through paternal leakage, implying that the paternal mitochondria are not always extinguished during egg fertilization [62]. For instance, in *Drosophila melanogaster* Meigen (Diptera: Drosophilidae), heteroplasmy due to paternal leakage reaches up to 14% in its sexually reproducing populations [63]. Variation in the mtDNA of a parthenogenetic species could indicate multiple sources of parthenogenesis [64]. For evolutionary studies, cytochrome oxidase subunit I (COI) is considered the most appropriate molecular marker among mitochondrial protein-coding genes [65], and has been widely used in Hemiptera [66,67,68].

It is speculated that *M. hellenica* was introduced into northern Greece from Turkey by the Romans and Byzantines [69], who are considered responsible for the artificial geographical range of the two primary hosts of *M. hellenica*, *P. halepensis* and *P. brutia* [70], since there are no references to the presence of *M. hellenica* in Greece during the prehistoric and classical eras [69]. Bouga et al. [37], who performed a COI mtDNA screening of individuals from four populations in Turkey, revealed a single haplotype. All the Turkish populations exhibited the same haplotype as that which is the most abundant in Greece, while one other, more geographically confined haplotype occurred in Greece. This vividly demonstrates the need for a multi-marker approach in future research efforts, including both nDNA and mtDNA markers, to accurately depict the pattern of intraspecific divergence. The results of the current research exhibit a high genetic affinity level between the populations of Greece and Turkey. If *M. hellenica* had invaded Greece from Turkey through multiple introductions, the genetic diversity in Greece would have reached the levels of its region of origin [44,71]. Given the presence of mainly one COI mtDNA haplotype throughout the sampling sites in both Greece and Turkey, it is most probable that the *M. hellenica* populations in the two countries share a common genetic origin. This has been suggested for other species, such as the parthenogenetic species *Dryocosmus kuriphilus* Yasumatsu (Hymenoptera: Cynipidae), which exhibited a single COI mtDNA haplotype, attributed to a single introduction from China to Europe [72].

The 15 Greek populations of *M. hellenica* analyzed in this study belonged to two COI mtDNA haplotypes. The predominant haplotype in Greece is identical to the single haplotype from four sites in Turkey exhibited by Bouga et al. [37], while the second haplotype found in this study was only present in three sites of northern Greece (Thessaloniki, Makriyalos, and Lefkada). The sites where the second haplotype was present, although they all belonged to northern Greece, did not exhibit geographic continuity, failing to explain a natural spread of the species. This can be attributed to dispersal through human activities, considering that *M. hellenica* is a principal contributor to the annual honey production in both Greece and Turkey [1,4] and, for this reason, it has been deliberately introduced into new regions of Greece [16]. Unfortunately, the human dispersal of *M. hellenica* impedes the interpretation of our results, further complicating the search for its origin.

### 4.2. Biological Traits of Marchalina hellenica Males

The exact reproduction strategy of *M. hellenica* remains unknown. Parthenogenesis is frequently observed in Hemiptera; however, scales demonstrate the most abundant variety of reproduction strategies [73], and the identification of the reproduction system of parthenogenetic species is considered a challenging task [74], with reproductive parasites and endosymbiotic bacteria further complicating the reproduction system’s identification [26]. For the first time in Greece, males, females, and 3^rd^-instar nymphs of *M. hellenica* were encountered at the same time of the year (January to late March) for two consecutive years (2021 and 2022), although males were found in low numbers compared to females, similarly to other coccids, which produce a sex ratio of 5%:95% (males:females) [75]. Male *M. hellenica* adults were encountered in Thessaloniki, where the second *M. hellenica* haplotype was present (GPS-HT2), indicating that males have a genetic effect on this population. The functionality of the male *M. hellenica* adults was not examined in this study through the inspection of mated females; however, the simultaneous emergence of 3^rd^-instar female nymphs, female adults, and male adults of *M. hellenica* is biologically sound, supporting the hypothesis of mating occurrence. The relatively high number of males during the two years indicates that some of the populations in northern Greece are facultatively parthenogenetic, whereas asexual lineages occur in southern Greece. Geographical parthenogenesis is observed in other insect species, such as *Clitarchus hookeri* (White) (Phasmatodea: Phasmatidae), in New Zealand [76], and *Coccus hesperidum* L. (Hemiptera: Coccidae), which all present one facultative parthenogenetic and one obligatory parthenogenetic lineage [77]. However, it is probable that *M. hellenica* reproduces sexually throughout its natural range, but has a low number of male individuals, as speculated recently [78].

In this regard, the Red Queen hypothesis, which has been applied to a wide range of organisms within Animalia [79,80,81,82], suggests that in coevolutionary struggles with natural enemies, the disproportionate attack of natural enemies on the most common phenotype could lead to the short-term coexistence of asexual and sexual populations [82,83,84]. Asexual reproduction would lead sexually reproduced natural enemies to become proficient at handling the defense mechanisms of a single clone, while their beneficiaries’ own capabilities would be continuously improved [85]. Furthermore, some species exhibit both sexual and parthenogenetic lineages on different hosts or in different geographical regions [24,74,86], with parthenogenetic populations often living within distinct ranges, such as marginal habitats, or at a higher latitude or altitude than sexual lineages [87,88,89]. Jensen et al. [90] suggested that sexual populations, usually found at the central part of the range of the infestation, act as sources of populations choosing asexual reproduction, which are found in the marginal regions of infestations. Consequently, mainland populations can be considered more biologically adapted than marginal populations, since they face the stress of a more complex set of natural enemies [85]. In the case of *M. hellenica*, several studies have examined the effect of the stress of *N. kartliana* on the scale’s populations, since it is the most abundant predator of *M. hellenica* [11,18]. Considering that *N. kartliana* has already been successfully used as a biocontrol agent against *M. hellenica* [17], it is most probable that it constitutes a major stress factor in the survival of *M. hellenica.* The sexual reproduction of *M. hellenica* and the abundance of *N. kartliana* in the same area [11] indicate that the reproductive strategy of *M. hellenica* can be explained by the Red Queen hypothesis, with mainland populations implementing sexual reproduction to counter the threat of *N. kartliana* to the survival of the population.

Asexual reproduction is a common feature among Hemipteran invaders, determining the success of invasions [91,92,93,94]. Considering that *M. hellenica* males exhibit a pattern of emergence, as suggested by this study, it is probable that an ongoing, or novel invasion of the species will be aided by the benefits of parthenogenesis, while the scale insect will also avoid the phenomenon of a genetic bottleneck due to facultative sexual reproduction, leading to DNA recombination. This emphasizes that *M. hellenica* constitutes a dangerous pest in the regions it has recently invaded.

## 5. Conclusions

In conclusion, the findings of this research provide new insights into the reproduction strategy of *M. hellenica* and its genetic affinity in Greece and Turkey. This contributes to the understanding of the establishment and ecology of this invasive species. However, this study also stresses the necessity for consistent investigation of the emergence of male *M. hellenica* individuals throughout not only its native habitat, but also the areas it has invaded, as described here, to better define the reproduction system of the species. Furthermore, additional research on the genetic variation throughout both Greece and Turkey, implementing a multi-marker approach, is needed to depict the pattern of intraspecific divergence of *M. hellenica* and determine its origin and genetic path.

## Figures and Tables

**Table 1:** COI mtDNA sequence of two haplotypes revealed in *M. hellenica* (Giant Pine Scale (GPS)) populations from Greece and Turkey.

Source	COI mtDNA Sequence
Turkey (GenBank HQ225738)	ATTAATACATCATTTTTCAATCCAAGAAGAAATGGAAGTCCA
Greece (GPS-HT1 GenBank OQ506006)	ATTAATACATCATTTTTCAATCCAAGAAGAAATGGAAGTCCA
Greece (GPS-HT2 GenBank OQ506007)	ATTAATACATCATTTTTTAATCCAAGAAGAAATGGAAGTCCA

## Data Availability

Data are contained within the article.
